# Gulf region smoking mortality trends and forecasts: A 30-year systematic evaluation of tobacco-attributable deaths

**DOI:** 10.18332/tid/210323

**Published:** 2025-11-27

**Authors:** Khalid A. Al-Regaiey, Fawaz Al-hussain, Turki Abualait, Muhammed Iqbal, Eman Mohammed Ali, Kaleem Maqsood, Shahid Bashir

**Affiliations:** 1 Department of Physiology, College of Medicine, King Saud University, Riyadh, Saudi Arabia; 2 Department of Neurology, College of Medicine, King Saud University, Riyadh, Saudi Arabia; 3 Department of Physical Medicine and Rehabilitation, College of Applied Medical Sciences, Imam Abdulrahman Bin Faisal University, Dammam, Saudi Arabia; 4 Department of Adult Neurology, Neuroscience Center, King Fahad Specialist Hospital - Dammam, Dammam, Saudi Arabia; 5 King Salman Center for Disability Research, Riyadh, Saudi Arabia; 6 Department of Biology, Lahore Garrison University, Lahore, Pakistan; 7 Neuroscience Center, King Fahad Specialist, Dammam, Saudi Arabia

**Keywords:** smoking-attributed mortality, Gulf Cooperation Council (GCC), tobacco-related disease, Global Burden of Disease, health policy

## Abstract

**INTRODUCTION:**

Tobacco use remains a major preventable cause of morbidity and mortality worldwide. Despite economic development and growing health system capacity, countries in the Gulf Cooperation Council (GCC) region face rising tobacco use and a shifting burden of smoking-attributed diseases. This study systematically analyzes age-specific and age-standardized smoking-related mortality trends across six Gulf countries Bahrain, Kuwait, Oman, Qatar, Saudi Arabia, and the United Arab Emirates over a 30-year period.

**METHODS:**

We extracted and analyzed smoking-attributed mortality data from the Global Burden of Disease 2021 dataset (1990–2019), including death rates per 100000 population for age groups 15–49, 50–69, and ≥70 years, and the age-standardized percentage of deaths due to smoking. We conducted descriptive trend analysis, heatmap visualization, correlation assessment, and linear projections to 2030.

**RESULTS:**

Bahrain and Kuwait exhibited the highest smoking-attributed mortality rates with 14.3% of all deaths in 2019, particularly among those aged ≥70 years, with death rates exceeding 1300 per 100000. In contrast, Saudi Arabia and Oman maintained relatively lower and stable rates. Strong correlations were observed between mid-life and elderly smoking mortality (r about 0.97), while slightly lower in the ≥70 years group (r about 0.85). Projections indicate a highest burden increase in Bahrain (about 13.5%) and slight increases in Qatar and UAE (about 9%, each) by 2030 without policy intensification.

**CONCLUSIONS:**

Despite regional advances in tobacco control, the burden of smoking-related mortality remains high in parts of the Gulf, especially among older adults. Immediate, targeted interventions particularly for middle-aged smokers are necessary to prevent further escalation. These findings support prioritizing tobacco cessation and surveillance as part of GCC public health strategies.

## INTRODUCTION

Tobacco use remains one of the leading preventable causes of death and disability worldwide. Despite considerable global progress in tobacco control, more than 8 million deaths each year are still attributable to smoking-related diseases, including cardiovascular disease, cancer, and respiratory illnesses^1^. These deaths are not only tragic but also largely avoidable. Tobacco consumption represents a substantial burden on health systems, contributing significantly to both direct healthcare costs and broader economic losses due to productivity decline, premature mortality, and long-term disability^2-4^.

The Global Burden of Disease (GBD) Study 2019 identified smoking as the second-highest risk factor for attributable deaths globally, trailing only high systolic blood pressure^5^. In high-income and middle-income countries, smoking contributes to between 10% and 20% of all deaths annually^6,7^. While smoking prevalence has declined in many Western nations due to robust control measures, progress has been uneven across regions. Countries in the Gulf Cooperation Council (GCC) including Bahrain, Kuwait, Oman, Qatar, Saudi Arabia, and the United Arab Emirates (UAE), face unique challenges in tobacco control despite their rapidly modernizing healthcare systems and economic development^4,8,9^.

Although the Gulf states have historically reported lower overall smoking prevalence compared to global averages, recent trends suggest a rising public health threat. A significant proportion of adult males in countries such as Kuwait (39%) and Saudi Arabia (23%) are current smokers, and youth tobacco experimentation is on the rise across the region^10^. Compounding this issue is the popularity of shisha (waterpipe) smoking, which is often underestimated in its health consequences but has been shown to deliver comparable levels of carcinogens and carbon monoxide exposure as cigarettes^9,11^. Waterpipe use is particularly common among adolescents and young adults, often facilitated by cultural permissiveness and insufficient regulation^12^.

Despite formal commitments to global health frameworks, such as the WHO Framework Convention on Tobacco Control (FCTC), GCC countries demonstrate varying levels of policy implementation and enforcement. While Saudi Arabia and the UAE have taken major strides in introducing smoking bans in public areas and increasing tobacco taxes, other states like Bahrain and Kuwait continue to face significant barriers in curbing tobacco accessibility, affordability, and advertising^13^. Moreover, limited integration of smoking cessation services into primary healthcare and a lack of nationwide quitline infrastructure further hinder effective control.

A key aspect of understanding and addressing the tobacco epidemic lies in quantifying its health burden, particularly in terms of smoking-attributed mortality. Smoking contributes to a range of diseases that typically manifest over long latency periods, including chronic obstructive pulmonary disease (COPD), ischemic heart disease, cerebrovascular disease, and several cancers^14-16^. This delayed impact underscores the importance of longitudinal data analysis to accurately assess trends, identify at-risk populations, and evaluate the effectiveness of public health interventions.

Despite increasing attention to smoking prevalence, data on smoking-attributed mortality over time in the Gulf region remain limited. National reports often lack age-specific trends or temporal analysis of disease burden. While the GBD study offers robust estimates, these have not been fully explored in a Gulf-specific context. This study addresses this gap by analyzing age-stratified smoking-attributed mortality across the six GCC countries from 1990 to 2019 using GBD 2019 data. Findings aim to highlight national disparities, age-group burdens, and the impact of tobacco control efforts.

## METHODS

### Study design and objectives

This study employed a retrospective ecological analysis using secondary data from the GBD dataset to quantify and analyze the burden of smoking-attributed mortality in the GCC countries, namely Bahrain, Kuwait, Oman, Qatar, Saudi Arabia, and the UAE, from 1990 to 2019. The primary objective was to examine age-specific and age-standardized smoking-related death rates, assess temporal trends, and generate future projections of mortality burden. The analysis aligns with the framework established by the GBD 2019 study, which provides comprehensive estimates of mortality and risk factor attribution for over 200 countries^17^.

### Data source

Data were extracted from a curated version of the GBD 2019 results, made publicly available through Our World in Data and the Institute for Health Metrics and Evaluation (IHME). The dataset includes age-standardized death rates and smoking-attributed mortality rates per 100000 population, disaggregated by age group and country. Smoking-attributed deaths were estimated based on comparative risk assessment (CRA) methodologies developed by the GBD collaboration, which combine data on exposure to tobacco use with relative risk estimates from meta-analyses and cohort studies^5^.

### The specific columns extracted

Data extracted included deaths attributed to smoking per 100000 people in three age brackets: 15–49, 50–69 years, and ≥70 years; the age-standardized percentage of all deaths attributed to smoking; and country, year, and ISO code identifiers for filtering and aggregation.

### Inclusion criteria and data handling

Only countries belonging to the GCC were included. Although the GBD includes data from 204 countries and territories, this study limited its scope to the six Gulf nations due to shared geographical, cultural, and economic contexts. The time period was restricted to 1990–2019 due to the availability and completeness of data across all included countries. The following variables were generated for analysis: total smoking-attributed death rate per country per year, computed as the sum of age-specific death rates (15–49, 50–69, ≥70 years); annual change rates and averages for descriptive analysis; and country-specific time-series trends and projections extending to 2030.

### Age group stratification

Three age groups were selected based on GBD conventions and WHO standards: 15–49 years (to capture early morbidity and emerging chronic disease risk), 50–69 years (to assess middle-aged populations where cumulative exposure typically manifests in elevated disease burden), and ≥70 years (to capture elderly populations with highest mortality risk from long-term smoking-related conditions such as COPD, lung cancer, and ischemic heart disease). This stratification allowed for assessment of how smoking-related deaths progress across the lifespan and facilitated evaluation of public health intervention timing.

### Outcome measures

The primary outcome of interest was the smoking-attributed death rate per 100000 population, by age group and country. The secondary outcome was the share of total deaths attributable to smoking, expressed as an age-standardized percentage.

This dual-level approach enabled both absolute and relative assessment of tobacco-related mortality. Age-standardized rates are particularly useful for international comparisons, as they adjust for demographic differences between countries^5^.

### Statistical and trend analysis

Descriptive statistics (mean, standard deviation, min, max) were calculated for each country across the study period. Time-series line plots were created to visualize trends in smoking death shares and age-specific rates. A heatmap analysis for Bahrain was conducted to illustrate longitudinal changes in age-stratified death rates. A correlation matrix was generated to explore relationships between age-specific death rates and the overall mortality share due to smoking, using Pearson’s correlation coefficients. Data preprocessing was conducted in Python (pandas and NumPy libraries), and visualizations were created using Matplotlib and Seaborn. Statistical analyses and projections were executed using *scikit-learn* for linear regression modeling.

### Projection modeling

To forecast smoking-attributed mortality shares beyond 2019, a linear regression model was fitted to historical data for each country. The model used ‘year’ as the independent variable and ‘age-standardized share of smoking-attributed deaths’ as the dependent variable. Projections were extended to the year 2030, assuming continuation of existing trends without major policy disruption. Model performance was assessed using R^2^ values, and 95% confidence intervals were calculated to assess uncertainty. While more complex models (e.g. ARIMA, exponential smoothing) exist, linear regression was selected for interpretability and consistency with previous GBD trend analyses^18^.

### Ethical considerations

As this study relied exclusively on secondary data from publicly available global databases, no human subjects were involved and no ethical approval was required. All data sources are anonymized and compliant with open data sharing protocols.

## RESULTS

This study systematically analyzed age-standardized and age-stratified smoking-attributed mortality across six Gulf countries, Bahrain, Kuwait, Oman, Qatar, Saudi Arabia, and the UAE, from 1990 to 2019. The analysis utilized smoking-attributed death rates per 100000 population across age groups (15–49, 50–69, and ≥70 years), as well as the percentage of all deaths attributable to smoking. Given the lack of gender-disaggregated data, all figures represent aggregate population-level findings.

### Temporal trends in smoking-attributed mortality share

**Figure 1:**
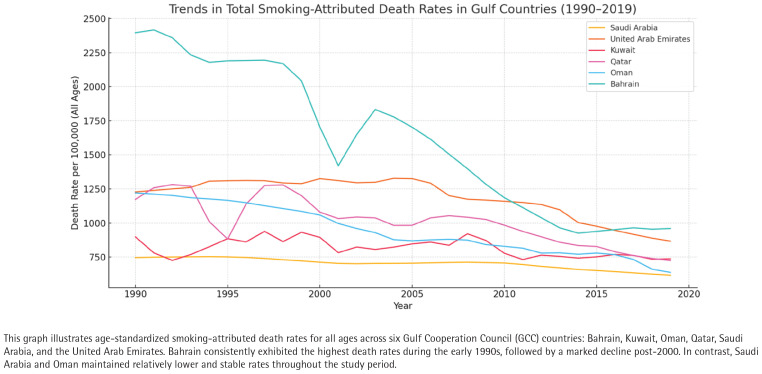
Trends in total smoking-attributed death rates per 100000 population in Gulf Countries (1990–2019); age-standardized rates for Bahrain, Kuwait, Oman, Qatar, Saudi Arabia, and the United Arab Emirates, based on the Global Burden of Disease 2019 dataset

**Figure 2:**
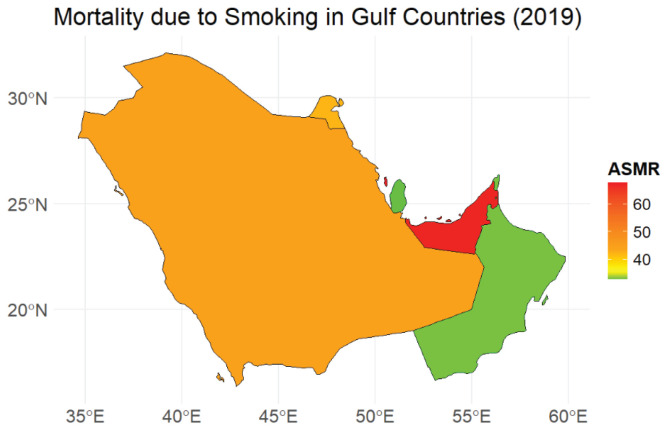
Comparison of smoking-attributed death rates per 100000 population in Gulf Countries (2019); Age-standardized rates for Bahrain, Kuwait, Oman, Qatar, Saudi Arabia, and the United Arab Emirates, based on the Global Burden of Disease 2019 dataset

Over the three-decade span, the share of total deaths attributable to smoking showed country-specific variation (Figure 1). Bahrain consistently exhibited the highest burden, with smoking accounting for more than 17% of all deaths in 1990, tapering slightly to 14.3% in 2019. Kuwait followed, starting at 10.8% and slowly declining to around 9.2% by 2019. In contrast, Oman and Saudi Arabia demonstrated more favorable trends, maintaining a relatively lower and stable share, 7.5% in Saudi Arabia and 8.0% in Oman, respectively. Qatar and the UAE maintained moderate values throughout, with minor fluctuations but no substantial upward trends (Figure 2).

These trends underscore disparities in public health policy implementation and tobacco control effectiveness. While Bahrain and Kuwait’s persistent high percentages suggest enduring challenges, Oman and Saudi Arabia’s stability may reflect better health surveillance systems, awareness campaigns, or taxation policies that have impacted smoking behavior over time.

### Age-stratified smoking death rates across the region

**Figure 3:**
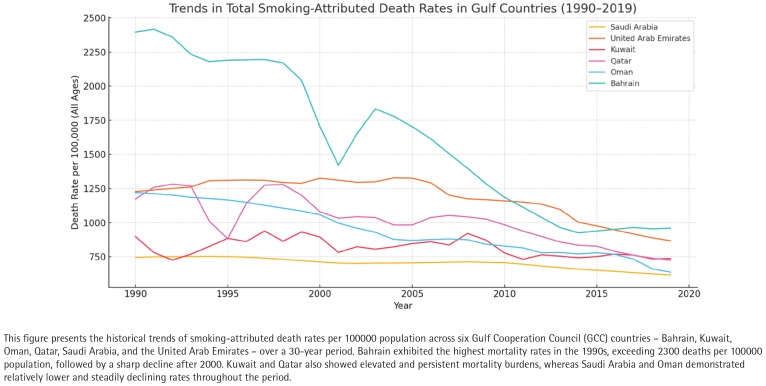
Trends in total smoking-attributed death rates per 100000 (all ages) in Gulf Countries (1990–2019); raw rates for Bahrain, Kuwait, Oman, Qatar, Saudi Arabia, and the United Arab Emirates, based on the Global Burden of Disease 2019 dataset

A deeper examination by age group revealed a predictable increase in death rates with advancing age across all countries (Figure 3). The age group 15–49 years exhibited relatively low smoking-related mortality rates, generally ranging between 5 and 20 deaths per 100000 population, with Bahrain and Kuwait showing the highest figures in this demographic. For instance, Bahrain averaged 10.16/100000, while Kuwait averaged 12.58/100000 in this younger cohort.

In the age group of 50–69 years, death rates increased substantially. Bahrain again ranked highest, with a mean of 236.65/100000, followed by Oman (232.14/100000) and Saudi Arabia (194.19/100000). This suggests a delayed manifestation of tobacco-related chronic illnesses such as ischemic heart disease, stroke, tobacco related chronic illnesses such as ischemic heart disease, stroke, COPD and certain cancers.

The age group of ≥70 years bore the most significant burden, with death rates exceeding 1300/100000 in Bahrain and 850/100000 in Qatar. Kuwait, Oman, and the UAE also showed elevated figures, averaging between 630 and 700/100000. Notably, Saudi Arabia maintained a markedly lower rate (497.04/100000) compared to its neighbors, which may be attributed to differences in population age structure, health system accessibility, or cohort smoking prevalence.

These age-specific observations are further summarized in Table 1, which shows the average mortality burden for each country over the 1990–2019 period. Bahrain exhibited the highest average total smoking death rate (1608.74/100000) and the highest share of smoking-attributed deaths (13.9%), while Saudi Arabia had the lowest (703.21/100000; 7.58% of deaths attributed to smoking).

**Table 1:** Average smoking-attributed mortality rates by country and age (1990–2019); average deaths for Bahrain, Kuwait, Oman, Qatar, Saudi Arabia, and the United Arab Emirates, based on the Global Burden of Disease 2019 dataset

*Country*	*Mean deaths 15–49 years*	*Mean deaths 50–69 years*	*Mean deaths ≥70 years*	*Mean percent of total deaths*	*Mean total death rate*
Bahrain	10.16	236.65	1361.94	13.90	1608.74
Kuwait	12.58	166.12	636.97	14.44	815.67
Oman	9.15	232.14	703.61	8.03	944.90
Qatar	7.71	152.97	854.23	8.84	1014.92
Saudi Arabia	11.98	194.19	497.04	7.58	703.21
United Arab Emirates	14.74	234.8	939.2	10.52	1188.74

### Country-specific trajectories and heatmap analysis

**Figure 4:**
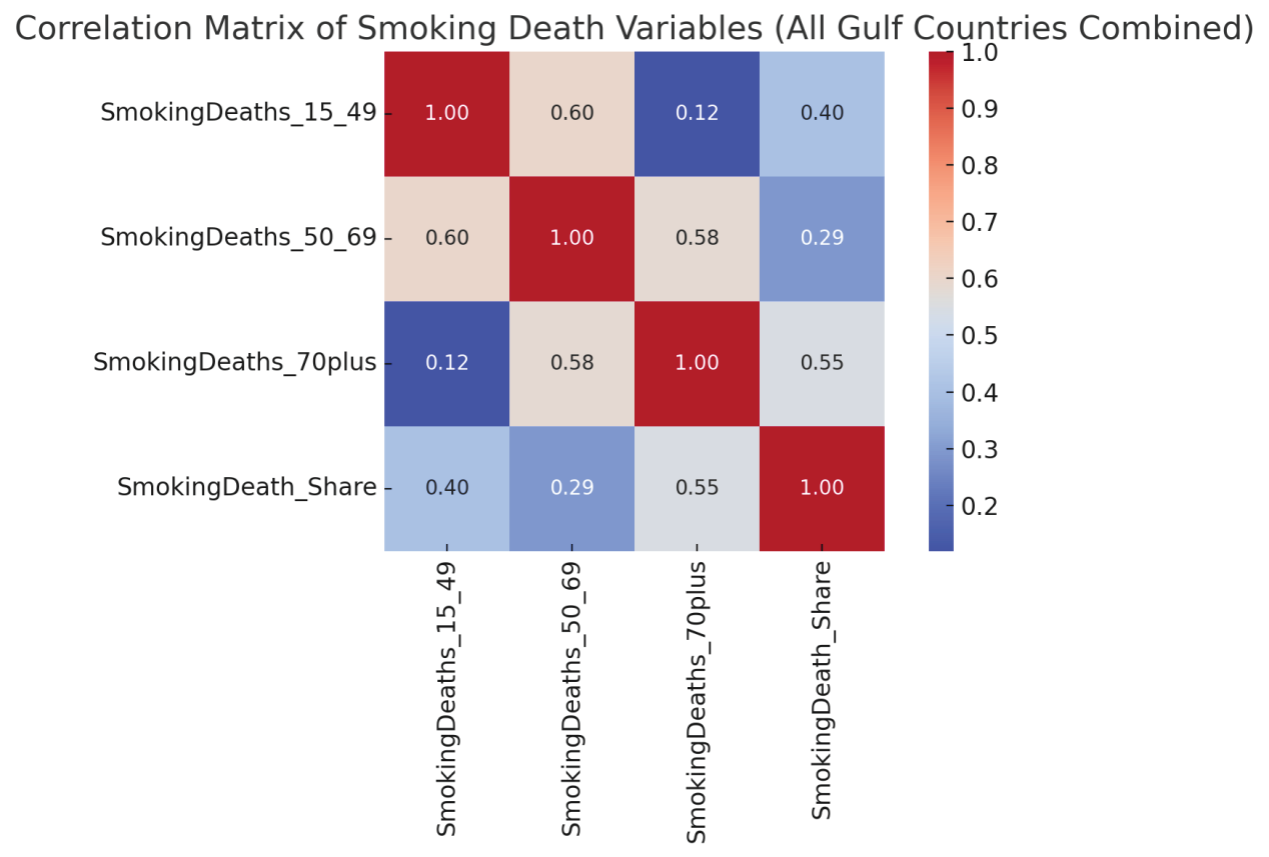
Correlation matrix of smoking-attributed deaths by age group and death share in Gulf Countries (1990–2019); age-standardized rates for Bahrain, Kuwait, Oman, Qatar, Saudi Arabia, and the United Arab Emirates, based on the Global Burden of Disease 2019 dataset

A detailed heatmap analysis of Bahrain’s smoking mortality by age group from 1990 to 2019 (Figure 4) revealed persistently high death rates among individuals aged ≥70 years, particularly in the early 2000s when values exceeded 1900/100000. The age group of 50–69 years also showed an initially high burden, peaking in the 1990s before declining modestly over time. However, no major improvements were observed in the elderly age group, emphasizing the long-term consequences of cumulative smoking exposure.

This heatmap provides visual confirmation of the need to address long-latency diseases that emerge after decades of tobacco use. It also highlights the limited impact of more recent tobacco control interventions on older generations who began smoking prior to the implementation of regulations.

### Correlation analysis of smoking mortality variables

Correlation analysis across the entire Gulf dataset demonstrated a very strong positive relationship (r about 0.97; 95% CI: 0.93–0.97) between smoking-attributed mortality in the 50–69 and ≥70 years age groups, reinforcing the notion that middle-age smoking patterns are strong predictors of elderly mortality burden. Additionally, the overall percentage of deaths due to smoking was closely correlated with the mortality rates in the ≥70 years group (r about 0.85; 95% CI: 0.68–0.92), suggesting that most of the tobacco-related burden is concentrated in older age demographics. This statistical insight emphasizes that population-level tobacco control will only meaningfully reduce the total burden of smoking-related mortality when middle-aged smokers are effectively targeted, given their likely progression into the higher risk elderly demographic.

### Projected mortality trends (2020–2030)

**Figure 5:**
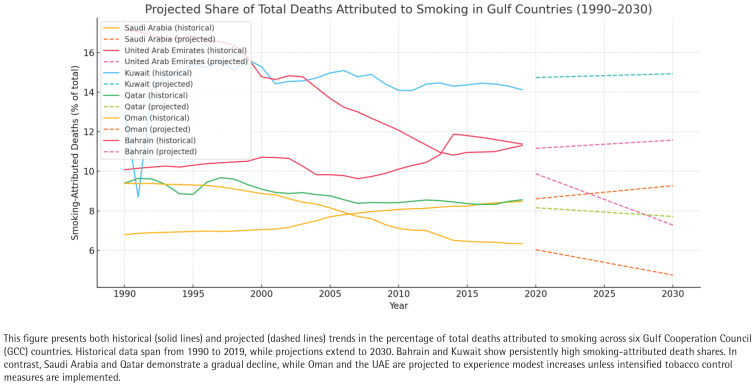
Projected share of total deaths attributed to smoking in Gulf Countries (1990–2030); age-standardized rates for Bahrain, Kuwait, Oman, Qatar, Saudi Arabia, and the United Arab Emirates, based on the Global Burden of Disease 2019 dataset

To understand the future trajectory of smoking-related mortality, linear regression models were developed for each country based on the historical trend in the share of total deaths due to smoking. Projections to the year 2030 revealed persistent disparities among countries (Figure 5).

If current trends continue, Bahrain will likely maintain the highest share of smoking-attributed deaths (about 13.5% by 2030). Saudi Arabia and Oman are projected to remain low and stable, below 8%. Qatar and the UAE are expected to slightly increase, reaching around 9%. Kuwait is projected to stabilize between 9% and 10%, depending on intervention uptake. While linear models provide a general direction, they do not account for possible future disruptions like changes in smoking prevalence, taxation, anti-tobacco campaigns, or healthcare reforms. Nonetheless, the projections offer valuable insight into which countries may require more urgent policy attention to avert future increases in smoking-related mortality.

## DISCUSSION

This systematic analysis of smoking-attributed mortality in the Gulf region over three decades provides valuable insight into the temporal, age-specific, and inter-country variation in tobacco-related health outcomes. The findings highlight both progress and ongoing challenges in reducing the burden of disease due to smoking, with substantial implications for public health strategy, healthcare resource allocation, and long-term policy planning.

Our study found that while some GCC countries, such as Saudi Arabia and Oman, have maintained relatively low and stable smoking-attributed mortality rates, others – particularly Bahrain and Kuwait – continue to experience a disproportionately high burden. Bahrain consistently had the highest age-standardized percentage of deaths attributed to smoking (13.9% on average), while Saudi Arabia maintained the lowest (7.6%)^4,6^.

Age-stratified analyses revealed that the burden of smoking rises sharply with age. Death rates in the ≥70 years age group far exceeded those of younger populations, reflecting the long latency between smoking initiation and the onset of chronic diseases such as chronic obstructive pulmonary disease (COPD), cardiovascular disease, and smoking-related cancers. This pattern is consistent with global epidemiological data showing that tobacco exposure during youth leads to disease manifestation primarily in older adulthood^4,8,19^.

Moreover, projections to 2030 suggest a concerning scenario for countries such as Bahrain and Qatar, where the smoking-attributed share of total deaths is projected to remain high or increase slightly if current trends persist. These findings emphasize the urgent need for intensified and sustained tobacco control measures, particularly among middle-aged and elderly cohorts.

### Regional comparison and interpretation

The GCC countries share many sociopolitical and economic similarities, yet display notable variation in smoking mortality trends. This divergence likely reflects differences in national health policies, tobacco taxation enforcement, prevalence of shisha use, public awareness levels, and healthcare system capacity.

For example, Bahrain and Kuwait, which reported the highest smoking-attributed death shares, have faced historical challenges in reducing tobacco consumption. In Bahrain, despite ratifying the WHO FCTC in 2004, studies have shown that implementation gaps in taxation, public education, and enforcement persist^13^. Kuwait, which has one of the highest smoking prevalence rates among adult males in the region (approximately 39%)^10^, also lags in comprehensive cessation services and restrictions on tobacco advertising.

In contrast, Saudi Arabia and Oman have implemented stricter tobacco control policies in recent years, including graphic warning labels, bans on smoking in public spaces, and price increases through taxation^4,8,20^. These measures may help explain their relatively lower smoking-attributed mortality burden. However, the persistently high rates in older age groups indicate that these policies may not fully address the needs of aging populations who began smoking decades earlier.

Furthermore, countries such as Qatar and the UAE present intermediate cases. While they have launched successful anti-smoking media campaigns and school-based programs, the uptake and long-term impact of these initiatives remain to be seen, particularly as youth vaping and shisha use increase^21^.

### Age-cohort dynamics and delayed policy impact

One of the central themes emerging from our analysis is the cumulative and delayed impact of smoking exposure, particularly in older adults. Death rates among individuals aged ≥70 years were consistently 5–10 times higher than those in younger age groups. This is consistent with longitudinal cohort studies from North America and Europe, which show that individuals who smoke heavily over decades experience a two- to three-fold increased risk of premature mortality^22^.

This finding has two important policy implications. First, current interventions focused only on youth prevention may be insufficient to reduce national smoking mortality in the short-term. While preventing initiation is critical, it will take decades for its benefits to manifest in reduced elderly mortality. Second, comprehensive cessation services targeting middle-aged and older adults must be prioritized. These include behavioral counseling, pharmacotherapy (e.g. nicotine replacement therapy, varenicline), and integration of cessation services into primary healthcare^1^.

It is also important to note that the legacy of tobacco industry influence and shisha culture in the Middle East may undermine policy efficacy. Shisha (waterpipe) smoking, while often perceived as less harmful than cigarette smoking, is associated with comparable levels of toxicant exposure and disease risk^11^. Studies from the region have shown that shisha use is particularly prevalent among young adults and even women, contributing to a hidden burden of smoking-related disease that may not be fully captured in current data^23^.

### Projected trends and future outlook

Our projection analysis revealed divergent future trajectories among Gulf countries. If linear trends continue, Bahrain is expected to maintain one of the highest smoking-attributed death shares through 2030, while Saudi Arabia and Oman remain below 8%. These projections serve as valuable early warning indicators. Without further public health investment, middle-income countries with high baseline prevalence – like Bahrain and Kuwait – may experience worsening disease burdens despite progress elsewhere. This is in line with broader findings from the GBD 2019 study, which reported that smoking remains among the top five leading risk factors for mortality globally, particularly in high-income and middle-income settings^5^. To counteract projected increases, aggressive implementation of FCTC provisions must be a regional priority. This includes enforcing plain packaging, banning flavored tobacco products, strengthening school-based interventions, and providing universal access to cessation aids. Policies should also address the emergence of electronic nicotine delivery systems (ENDS), which are gaining popularity among youth and may serve as a gateway to lifelong nicotine dependence^24^.

### Limitations

This study is limited in the following ways. First, there is a lack of data on gender and thereby it is not possible to examine the outcomes in sex-specific mortality trends, which are significant since the prevalence of smoking among males in the Gulf is greater than in females. The analysis was also unable to take into account young populations, which would have identified possible secondary effects of passive smoking or exposure *in utero* ^25^. Moreover, the estimates on mortality involved GBD and this could be underreported or misclassified in countries whose health information systems are weak. The linear regression model which is applied to projections presupposes a constant state of trend, neglecting non-linear effects of policy changes including those on emergent tobacco trends, including e-cigarette usage and dual smoking. Finally, the trends may be impacted by unmeasured social and cultural confounding and the use of aggregate data may lose significant population-level relationships. Future research must include trending information and research to refine the models to make up these gaps. Future studies should explore scenario-based modeling approaches that incorporate policy interventions, demographic aging, and behavioral trends. Nonetheless, the study’s use of harmonized global data and rigorous analytical methodology provides a valuable foundation for comparative analysis and policy development in the GCC context.

## CONCLUSIONS

The burden of disease attributable to smoking in the Gulf region remains high, particularly in older populations and in countries like Bahrain and Kuwait. While some nations have made progress in curbing tobacco use, others face persistent challenges linked to cultural, economic, and policy-related factors. Without intensified intervention, current trends predict a continued high mortality burden from smoking-related illnesses. This study underscores the urgent need for targeted, multi-tiered tobacco control strategies adapted to the demographic and epidemiological profile of each country.

## Data Availability

The data supporting this research are available from the query tool of Global Health Data Exchange ( http://ghdx.healthdata.org/gbd-results-tool ).
